# Word learning tasks as a window into the *triggering problem* for presuppositions

**DOI:** 10.1007/s11050-024-09224-5

**Published:** 2024-10-14

**Authors:** Nadine Bade, Philippe Schlenker, Emmanuel Chemla

**Affiliations:** 1 Linguistics Department, University of Potsdam, Karl-Liebknecht-Strasse 24–25, Potsdam, 14476 Germany; 2 Institut Jean Nicod (EHESS, CNRS), Département d’Etudes Cognitives, Ecole Normale Supérieure, PSL University; New York University, 29 Rue d’Ulm, Paris, 75005 France; 3 Laboratoire de Sciences Cognitives et Psycholinguistique (EHESS, CNRS), Département d’Etudes Cognitives, Ecole Normale Supérieure, PSL University, 29 Rue d’Ulm, Paris, 75005 France

**Keywords:** Presuppositions, Triggering, Word learning, Projection

## Abstract

In this paper, we show that native speakers spontaneously divide the complex meaning of a new word into a presuppositional component and an assertive component. These results argue for the existence of a productive triggering algorithm for presuppositions, one that is not based on alternative lexical items nor on contextual salience. On a methodological level, the proposed learning paradigm can be used to test further theories concerned with the interaction of lexical properties and conceptual biases.

## Introduction

Presuppositions are non-literal, “non-at-issue” parts of meaning which are traditionally considered preconditions for felicitous utterances. Under a traditional semantic view, they are associated with certain lexical units, called *presupposition triggers*. An example of a presupposition trigger is the verb *stop*. A clause containing *stop* presupposes the existence of an initial state and asserts a change of state. The sentence in (1), for example, presupposes that there was a time when Peter smoked (=initial state) and asserts that Peter does not smoke now (=change of state).

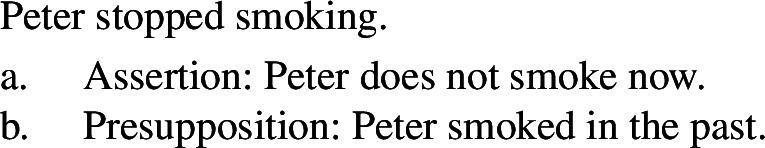

Two properties of presuppositions that have featured prominently in the literature are their sensitivity to common ground knowledge and their projection behavior. Regarding the first property, the observation is that sentences with presuppositions usually cannot be uttered felicitously without interlocutors agreeing that the presupposition holds, i.e., without shared knowledge of the presuppositional content (Stalnaker 1977, 2002). Second, presuppositions—as opposed to assertions—remain stable under operators such as negation and antecedents of conditionals (Karttunen 1973). For example, as shown in (2-b), the inference in (1-b) still holds when the sentence is negated; see (2). By contrast, (1-a) does not follow when the sentence is negated, as shown by (2-a).

(2)
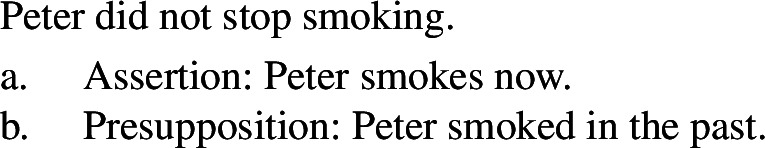

Both of these properties have been used as diagnostics to determine whether a certain component of meaning is a presupposition. If a sentence requires that all participants in the conversation are committed to the truth of one of its meaning components, it is an indication that this part of meaning is presupposed. If the part of meaning remains stable under negation and other operators (antecedent of conditionals, questions, as in the “family of sentences”-test; see Chierchia and McConnell-Ginet 2000) projects out of these environments, this presents further evidence for presuppositional status. The theoretical discussion of these properties has become more complex due to different presupposition triggers displaying these characteristics to varying degrees (Simons 2001; Romoli 2011). Data from experimental and cross-linguistic research (Tiemann 2014; Tiemann et al. 2015; Simons et al. 2016; Domaneschi et al. 2018, 2022; Tonhauser et al. 2018; Degen and Tonhauser 2021) show that projection behavior and the felicity of out-of-the blue uses of presuppositions are affected by a multitude of factors. This includes the lexical properties of the trigger itself, as well as contextual factors such as information structure and focus placement.

The current paper is concerned with the theoretical issue of how presuppositions arise, that is, the question what parts of meanings become presuppositions. The literature, motivated by the cross-linguistic stability of the presupposition/non-presupposition divide, argues for the need for an explicit rule that specifies which entailments of elementary expressions should be treated as presuppositions (Abrusán 2011; Schlenker 2021b).

More recent arguments for such a productive “triggering algorithm” come from the study of gestures and visual animations (Tieu et al. 2019; Schlenker 2021a,b). The crucial aspect here is that some of these expressions (especially visual animations) could not have been seen before. Their bivalent content can presumably be understood without prior exposure thanks to iconic rules, e.g., the kind of projection-based semantics advocated in Greenberg (2013) in the semantics of pictures.^1^ On the assumption that iconic rules only yield a bivalent content, however, it is striking to find that these unseen expressions can trigger presuppositions (as confirmed by projection tests). This is most compatible with the existence of a general triggering algorithm.

Productive triggering has never been demonstrated for words. We propose a new experimental paradigm in which bivalent content is induced through exposure to usage conditions of non-words (the lab version of the normal lexical acquisition process).

In Experiment 1, we find that presuppositions are obtained after very little, neutral exposure to a new word. Experiment 2 shows that exposure to negative sentences, an *a priori* abundant and clear-cut type of evidence for presuppositions, is not able to reverse what default presuppositions are chosen for a new change-of-state verb. Experiment 3 confirms the presuppositional status of the phenomenon we observed, by looking at typical projection tests in quantified sentences. All three experiments together speak to the success of the new methodology used to investigate what factors play a role in presupposition triggering.

## Current theories

Different approaches have been taken to the triggering problem for presuppositions. For illustration, consider again a change-of-state verb, such as *stop*. The observation to be accounted for is that the initial state shows behavior in line with an analysis as a presupposition, whereas the ensuing state appears to be part of the assertive component. What predicts this pattern under the different approaches? Existing theories highlight different factors for the observed division of labor between assertion and presupposition.

### Hybrid approaches with contextual information

#### Contextual alternatives

One type of theory offers a hybrid approach where both lexical and contextual information play a role in presupposition triggering. These accounts relate presuppositions to lexical alternatives (Abusch 2002, 2010; Romoli 2011; Chemla n.d.), especially for a group of so-called “soft” presupposition triggers, such as *stop*, *win* and *know*. The presuppositions of these triggers have been argued to behave more like assertions in the sense that they can be new information and are sometimes visible to operators like negation and antecedents of conditionals (do not project). The lexical alternatives of these softer triggers may or may not be activated depending on the local and global context. Accordingly, a contextual component is built into alternative-based views on presupposition triggering.

In her alternative-based account for a subset of presupposition triggers Abusch (2002) relies on the alternatives in (3).^2^

(3)
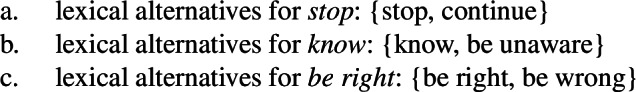

Abusch proposes that these triggers come with a conversationally triggered presupposition that the disjunction of their alternatives is true, see (4), for example.

(4) . The presupposition of *Peter stopped smoking* is
  ‘Peter stopped smoking or Peter continued smoking.’ ⇒ John smoked in the past

Under alternative-based views, the triggering problem is reduced to the question of explaining why a trigger has the alternatives it has and when they are relevant. In its simplest version, it depends on the presence of lexical alternatives and the contextual factors for their contextual activation, see more discussion in Sect. 5.

#### Contextual questions

A second type of contextual view relates the division of labor between asserted and presupposed content back to focus and contextual questions (Simons et al. 2011, 2016; Tonhauser et al. 2018). Alternatives thus indirectly play a role under this view but are assumed to arise through contextual information and information structure rather than access to lexical scales. This view subsumes presuppositions under “non-at-issue”-content, which is defined as meaning which does not directly address the current question (CQ) under discussion, or a relevant sub-question. The assumption is that the projection behavior of presuppositions is rooted in that property, not lexical properties of certain words. The claim is that all meaning components which are “at-issue” are visible to operators, and thus do not project, whereas “non-at-issue” meaning is not visible to these operators and, as a result, projects.^3^ For example, due to focus on *discovers* in (5-b), the at-issue part of the sentence is whether the T.A. becomes aware of plagiarism, whereas the fact that there was plagiarism seems to be a given. As a result, the former component of meaning does not project from the antecedent of the conditional, whereas the latter does. The same does not hold (or holds to a lesser degree) for (5-a). With focus on the verb, both parts could be at issue, and thus become visible to operators (hence they do not project). As a result, the if-clause could be interpreted as ‘if your work is plagiarized and the T.A. discovers it…’.

(5)
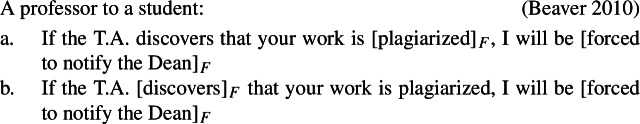

Under this variant of a contextual view, the triggering problem is reduced to explaining what content is more likely to be “non-at-issue”/not addressing the CQ and thus becomes projective.

### Conceptual approaches

A second type of approach assumes that certain conceptual biases play a role in presupposition triggering. Theories taking this approach assume a productive triggering mechanism to be active.

#### Presupposing information that is not about the topic time

One such a theory is offered by Abrusán (2011, 2012). Her account is only concerned with verbal presupposition triggers and makes reference to the event time of the matrix predicate. Specifically, she predicts that those entailments that are *not* about the topic time end up presupposed. For *stop*, the entailment in (6-b) of the sentence in (6) is about the topic time, whereas the entailment in (6-c) is about a time before the topic time. (6-c) is thus correctly predicted to come out as presuppositional.

(6)
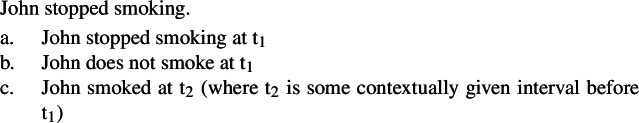

It is important to note that Abrusán’s view, just as the contextual views described above, assumes that those entailments become presuppositions which the sentence is not mainly *about*. Her theory makes explicit what concept aboutness is rooted in, however: namely, temporal information. Using topic times and the notion of aboutness is straightforward for change-of-state verbs expressing temporal relations. The theory requires additional machinery and stipulations for factive verbs.

#### Presupposition information that is likely to be pre-existing knowledge

Schlenker (2021b) discusses potential over- and undergeneralization problems with this approach, and suggests another conceptual approach based on epistemic preconditions. Informally speaking, his view is based on intuitive access to the probability of pre-existing knowledge of the presupposition upon learning the assertion. The algorithm he proposes predicts that if *p* is an entailment of a propositional expression *E* and *p* meets a certain probability threshold for being an antecedent belief (is typically antecedently believed) upon learning *E*, *p* will be treated as a presupposition. Applied to the case of change-of-state verbs, the initial state usually comes out as presupposed under the assumption that one is more likely to have antecedent beliefs about past states than about present or future states. Even though we are subsuming Schlenker’s and Abrusán’s view under one approach working with conceptual biases for the purposes of the current paper, it is important to stress that they differ in important details. We come back to this point in the General Discussion in Sect. 5, and discuss how the new paradigm makes it possible to test their predictions directly and to distinguish between different conceptual views in the future.

We identified two main approaches to the triggering problem for presuppositions, highlighting different relevant factors for a meaning component to become a presupposition; its lexical status, especially its lexical alternatives, its contextual status, and its conceptual status. The experiments reported in Sect. 4 offer a new empirical perspective on how these different factors might interact and play a role in presupposition triggering. Rather than offering conclusive evidence for one or the other theory, the goal is to establish a new methodology which can be modified to test the predictions of individual theories directly.

## Previous experimental evidence—presuppositions in the CB paradigm

We will ask what, if any, presuppositions are associated with novel words. For this, we will rely on existing experimental paradigms used to study presuppositions. The covered box (CB) method is an experimental paradigm frequently used for investigating non-literal meaning, see e.g., Huang and Snedeker (2009) and subsequent work. The advantage of the method over a simple truth value judgment task is that participants are not forced to use a “false”-judgment for false implicatures or presupposition failure. This aligns with theoretical assumptions and previous findings that sentences whose implicatures or presuppositions are not true in the context are perceived as odd rather than false. Within the CB paradigm, participants are faced with an overt picture which makes the literal or non-literal meaning true versus false. They have as an alternative choice a covered box (a fully or partially covered picture) for which the truth value of all components of meaning in the given context is unknown. The task is to choose between the pictures given a sentence. Participants are instructed to choose the covered picture when the overt one does not meet the description of that sentence. In effect, by choosing the covered box, participants treat the sentence as “something other than true” in the context, but without having to explicitly categorize it using labels such as “false”, or more complicated ones such as “something other than true”. The assumption is that participants will definitely choose the covered box when the alternative’s assertion is false. Different considerations should play a role when the overt picture falsifies non-literal meaning, such as presuppositions and implicatures. This allows for a comparison between literal and non-literal meaning components.

One set of experimental studies on presuppositions using a covered box paradigm focus on presuppositions under negation and a comparison with direct and indirect scalar implicatures (SI). The theoretical underpinnings of these works revolve around the possible involvement of lexical alternatives in both cases (Romoli 2011), as well as what the role of entailed versus non-entailed content is. The findings are quite diverse, largely depending on the exact set-up of the study and the trigger tested.

Romoli and Schwarz (2015) find 75% covered box choices with violated presuppositions of *stop* under negation, same as for indirect scalar implicatures. Bill et al. (2018), however, find a slightly different pattern with a similar setup. They see CB rates of around 72% for direct SI, for indirect SI they find 50% CB choices, and for *stop* under negation only around 37%. In both studies, CB choices for positive sentences are at ceiling level for presupposition violation, similarly to false assertions. However, CB choices were slower when the presupposition was violated than when the assertion was false in the overt picture.

Another set of experiments employing CBs look at more complex projection cases involving quantifiers. We return to the projection properties of presuppositions in the scope of quantifiers in Sect. 4.3 below.

Zehr et al. (2016) use a CB task to investigate presuppositions of the achievement verb *win* (presupposition: participate) in the scope of the quantifier *none*. They find evidence for the existential reading (around 40% CB choices for images violating this reading) and the universal reading (around 25% CB choices violating this reading). They find no evidence for an existential reading for the same experiment with children. They speculate that the existential reading thus may have a different source or be a case of domain restriction.

Creemers et al. (2018) investigate presupposition by looking at covered box choices for triggers in the scope of the quantifiers *every* and *at least one*. They look at two types of triggers, the factive construction *is aware that* and the iterative particle *again*. For *at least one* they find: 75% CB choices for presuppositions being violated (existential and universal), with only a minimal difference between *again* and *is aware that*. They find almost no CB choices with violated universal presuppositions only. They argue that the existential reading is available for *at least one*. For *every* they find a much more mixed picture. They observe 50% CB choices with violated universal presuppositions. However, they can clearly identify groups of participants (accessing or not accessing the reading).

In sum, previous experimental evidence shows that CB tasks can track presuppositional behavior, especially when presuppositions are embedded, and can help distinguish between presupposed and asserted meaning. However, the findings also suggest that involving different types of embedding and dependent measures is crucial to empirical support of this distinction.

## Experiments

The logic behind all three experiments is as follows. In the first phase, we teach participants a new word by exemplifying its usage through animations. The meaning of the word, as shown by the animations, has two components. It shows an initial state of an object, and a change of state of that same object. In a second phase, participants are tested on their understanding of the word. They are given a sentence with the target word. They are then asked whether this sentence is a description of a situation explicitly represented via an animation, or if it is more likely to be the description of a different situation, represented by a static black box, as in the covered box paradigm. We test whether this newly learned word carries a presupposition, and if so, which part of its meaning is a presupposition.

### Experiment 1

In Experiment 1, participants were taught the meaning of a new word describing a complex event by being exposed to only positive instances of its usage conditions. The goal was to test whether participants would interpret any part of the meaning as presuppositional with limited input.

#### Method

##### Procedure and materials

The experiment started with very short and simple instructions, see (7).

(7) . In this experiment we will teach you a new word *wug*. Watch the animations carefully to see how it is used.

**Fig. 1:**
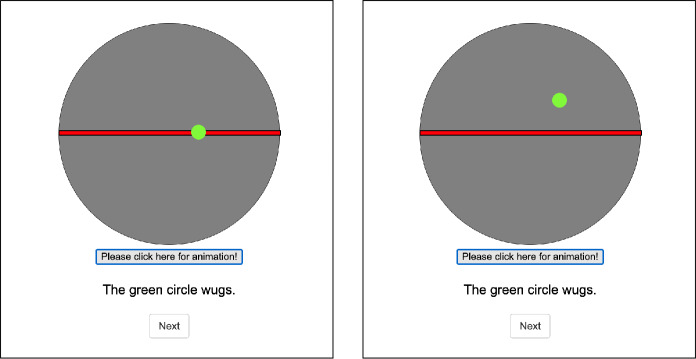
Screenshots of the visual animation at the initial (left) and final state (right) during the training phase

After the instructions the familiarization phase started, during which participants learned the meaning of *wug* by being presented with animations exemplifying its usage. We showed them several instances of a complex event consisting of two sub-parts *A* (initial state) and *B* (change of state). Specifically, participants saw a big gray circle, divided in half by a red horizontal line. There was a smaller green circle, which, when the animation was started through pushing a button, moved from a red line (=initial state) upwards to the upper gray half of the bigger circle (=change of state), see Fig. 1.

Participants could play the animation as many times as they wanted by pushing the “Please click here for the animation”-button. Below the animation, they saw a sentence describing the scenario and containing the new word *wug*, as in (8).

(8) . The green circle wugs.

Participants saw the “wugging” event together with the sentence in (8) four times in the training phase. The position of the green circle on the red line and how far upward it moved was varied for these four iterations. This variation was meant to prevent participants from assuming that “wugging” involves a specific position on the red line or a specific travel distance.

In the second phase of the experiment, we tested participants’ understanding of *wug* by showing them animations again. They falsified either none, one or both of its two meaning components (see Table 1). Animations were either presented together with the positive sentence in (9-a) or the negative sentences in (9-b).

**Table 1:** Animation names, types, and descriptions used in the experiment

Type	Animation
true control	from the red line, upward
target A	from the red line, not upward
target B	not from the red line, upward
false control (and pres. failure)	not from the red line, not upward

(9)

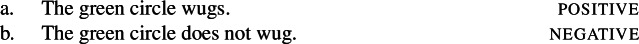

**Fig. 2:**
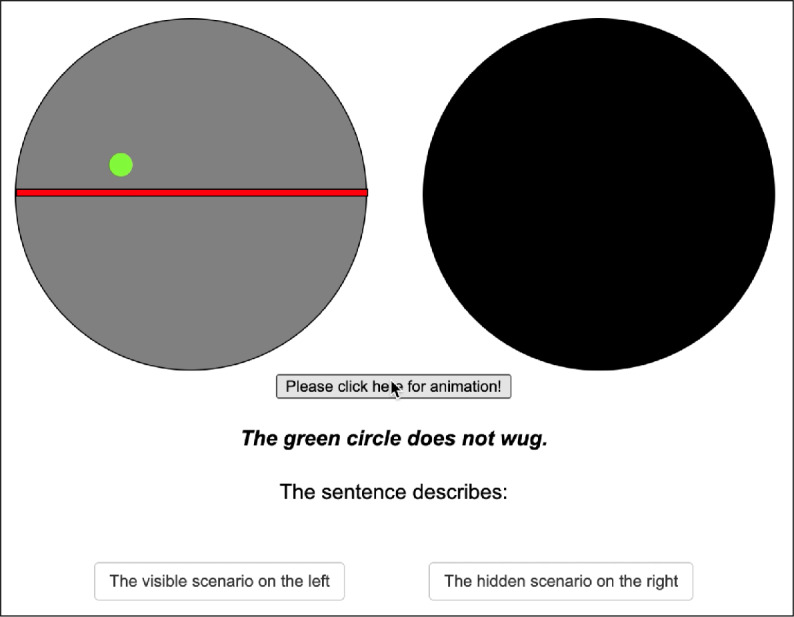
Screenshot of a trial during the testing phase

In addition to an overt animation having one of the properties in Table 1, participants were shown a covered animation (a black circle). Their task was to choose the animation they thought the sentence described. They were instructed to choose the covered one when they considered the visible one inappropriate. A screen shot of the testing phase is given in Fig. 2.

##### Design

We manipulated two factors within subjects: animation type with four levels (upward—from red, not upward—from red, upward—not from red, not upward—not from red) and sentence polarity with two levels (negative/positive). There were 4 target items per critical animation type, half of which were paired with negated sentences, the other half with positive sentences. There were furthermore 2 true and 2 false control animations, each of them appearing once with a negated sentence and once with an affirmative one. Furthermore, there were 8 filler animations paired with quantified statements (“{Every/None} of the green circles wug”) (=20 animations in total in the testing phase). Fillers were chosen to increase variability in the animations. They also served the purpose of allowing for a first look into whether the method is suitable for testing more complex sentence types. Trials were pseudo-randomized, with the first 4 trials being a true control or an A type target. The rest of the items followed in fully randomized order. The purpose of this pseudo-randomization was to confront people with clear cases in the beginning so they could get familiar with the procedure before responding to the critical cases.

##### Participants

We looked at the data from 49 native speakers of English who participated in the experiment via Prolific. None of the participants were excluded from the analysis of Experiment 1 as all of them responded accurately to the control animation they were trained on more than 50% of the time. Participants received what was (then) labeled “good” pay for their participation (7.50GBP/hour).

#### Hypotheses and predictions

The first research question addressed by our experiment was: is there a conceptual bias that determines the presuppositional status of a meaning component? To answer this question, we used the fact that presuppositions are not affected by negation in the same way as assertions. We checked two different hypotheses regarding default behavior in the absence of input that could decide between presupposition and assertion:

*Hypothesis 1a*: when learning a new word expressing a complex meaning M with meaning components M_*A*_ (initial state) and M_*B*_ (change of state), the meaning is construed as Assert(M_*A*_) and Assert(M_*B*_) (=“conjunctive interpretation”)
*Hypothesis 1b*: when learning a new word expressing a complex meaning with meaning components M_*A*_ (initial state) and M_*B*_ (change of state), the meaning is construed as Ps(M_*A*_) and Assert(M_*B*_)

Hypothesis 1b is consistent with conceptual views of presupposition triggering which predict the initial state to come out as presuppositional for conceptual reasons. Participants learn nothing about the information status, including the “at-issue” status, of the two meaning components during the familiarization phase, nor can they know anything about possible alternatives based on the learning phase. Hypothesis 1a is thus more compatible with a contextual/hybrid approach.

**Fig. 3:**
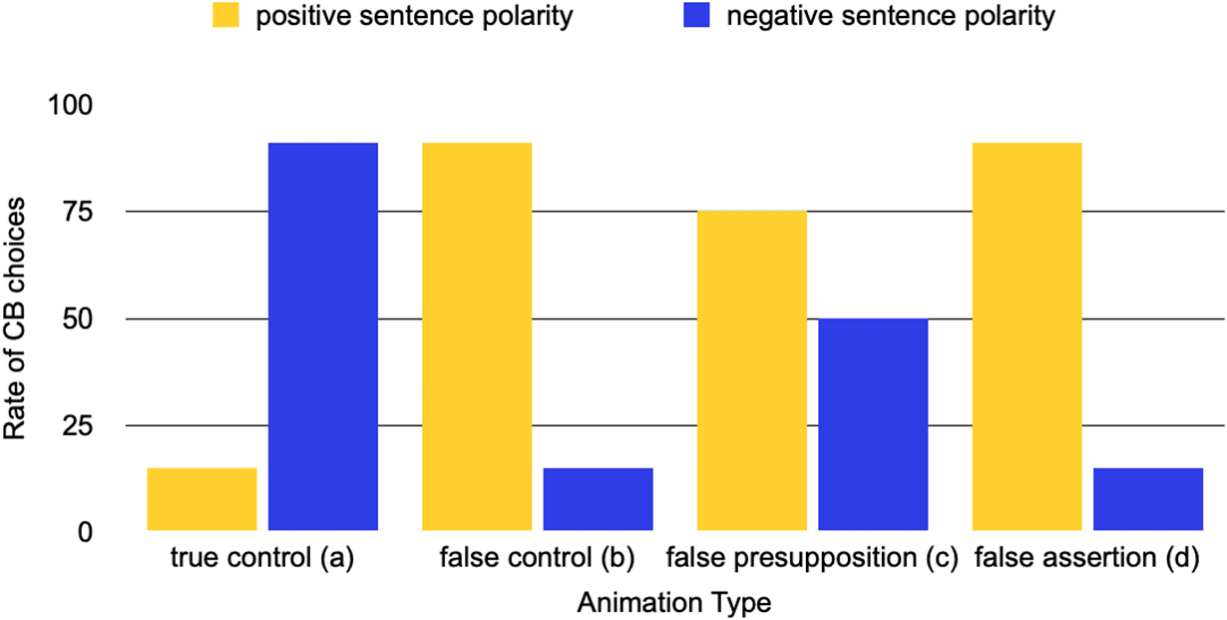
Predicted response patterns for when both meaning components are true = (a), for when both meaning components are false = (b), for when only presupposed meaning is falsified by the animation = (c), for when only asserted meaning is falsified by the animation = (d)

Figure 3 depicts the expected response patterns for different situations in which assertion or presupposition or both are falsified/verified by the animation. When both meaning components are true (= “true” controls), CB choices should be high with negation and low without (pattern (a)). When both meaning components are falsified by the animation (“false” controls), the mirror image should occur: with negation CB choices should be low, without negation high (pattern (b)). Theoretically speaking, if one of the meaning components is a presupposition, the “false” control is also a case of presupposition failure. Based on the results of previous CB studies investigating triggers whose presuppositions are also entailments, we expect at ceiling level CB choices for these cases (Zehr and Schwarz 2016). The main logic of the experiment is unaffected by this, since the animation condition that falsifies both meanings is not the one that critically distinguishes between the two hypotheses.

If only one meaning component is falsified by the animation, and that meaning is a presupposition, the pattern in (c) in Fig. 3 should be visible. That is, with and without negation, CB choices should be relatively high but not at ceiling. In contrast, if only one meaning is falsified, and that meaning is part of the assertion, we should see the pattern in (d) in Fig. 3, which is identical to the pattern expected for “false” controls. Given that the word is completely new to participants, it is hard to make predictions for exact rates of CB choices for presupposition failure. However, given previous results and the unique signature of presuppositions, the relevant comparison should be between negative and positive sentences. Specifically, there should be a starker contrast between negative and positive sentence polarity when the assertion is falsified by the animation than when the presupposed meaning is falsified.

How do these general predictions for response patterns relate to the hypotheses laid out above? If a mere conjunctive meaning is obtained (=H1a is correct), critical animation conditions should be sensitive to negation in the same way. That is, only a main effect of negation on critical target animations is predicted. Specifically, for both critical animation types “not upward from red” and “upward not from red” we expect the response pattern to be the mirror image of the “true” control animation condition (show pattern (b) in Fig. 3): with negation people should go for the overt picture, without negation for the covered animation.

On the contrary, if one of the meaning components (initial state) is treated as a presupposition and the other (result state) as an assertion (= H1b is correct), we predict an interaction between animation type and sentence polarity. Specifically, CB choices should be relatively high, irrespective of polarity of the sentence, for the target animation “upward not from red”. That is, we should see the pattern in (c) in Fig. 3. CB choices should be high with positive sentences and low with negative sentences for the target animation “not upward from red”. That is, that target animation should behave like “false” controls (pattern (b) in Fig. 3).

The predicted response patterns according to the two hypotheses are summarized in Table 2.

**Table 2:** Predictions for response patterns by critical animation conditions for Hypotheses 1a and 1b

Hypothesis	Prediction
H1a	target A/B = pattern (d) “false assertion”
H1b	target A = pattern (d) “false assertion”, target B = pattern (c) “false presupposition”

#### Analysis and results

We ran a linear regression analysis with generalized linear mixed-effects models using the lme4 package in the *R* programming language. We used model comparisons to establish whether our independent variables (animation type with 4 levels and sentence polarity with two levels) affect the dependent variable, which was the rate of choices of the covered box. We started with a full model estimating fixed effects of animation type and polarity, as well as by-participant and by-item varying intercepts and slopes for both factors, including their correlation. We used treatment coding with the reference level being “upward—from red” and negative sentence polarity. We reduced the random effect structure step-wise when the model did not converge. We always report the final models used for log likelihood ratio tests below.^4^

**Fig. 4:**
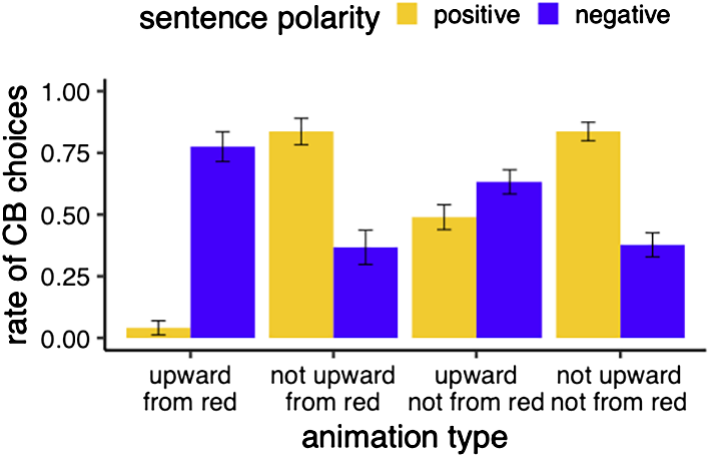
Exp. 1: Rate of covered box choices by polarity of sentence and animation condition. Error bars indicate the standard error

The results are shown in Fig. 4. Descriptively, we see that the two critical animation conditions react differently to sentence polarity. The responses to the animation falsifying upward movement look like the false control animations in that sentence polarity switches the response pattern compared to the true control. However, the animation condition where movement did not start from the red line shows a response pattern where CB choices were equally high irrespective of sentence polarity.

To look into Hypotheses 1a and 1b, we tested for an interaction of animation type with sentence polarity. To do so, we compared a model without interaction term (button_pressed ∼ negation + cond + (1 + negation | subjectId) and a model with the interaction term (button_pressed ∼ negation ∗ cond + (1 + negation | subjectId). The nested model comparison via log likelihood ratio tests revealed that the interaction term is justified ($\chi^{2}(3)=170.67$, *p*<.0001).

We looked at Bonferroni-corrected pairwise comparisons based on least square means using the emmeans package in *R*. It reveals that there is no difference between negative and positive sentences for the animation “upward - not from red”, but there is a significant difference between negative and positive sentences for the other three animation conditions, see Table 3.

**Table 3:** Exp. 1: Pairwise comparison output of contrasts based on least square means calculated with the emmeans package in *R*

cond	Contrast	*β̂*	SE	z.ratio	p.value
upward—from red	neg - pos	6.201096	1.102	5.626	*p* <.0001
not upward—from red	neg - pos	−3.346616	0.777	−4.307	*p* = 0.0003
upward—not from red	neg - pos	0.710891	0.544	1.308	*p* = 1
not upward—not from red	neg - pos	−3.298740	0.658	−5.015	*p* <.0001

#### Discussion

Overall, we see that CB choices increase with negated sentences for the animation condition that falsifies upward movement, but remain at the same level as with positive cases when animations falsify movement from the red line. Since sensitivity to polarity is diagnostic of assertions, these results suggest that upward movement is considered to be “at-issue”, asserted meaning, whereas moving from the red line shows behavior more in line with “non-at-issue”, presupposed meaning. Regarding the first main question we addressed, our findings are thus in line with Hypothesis 1b, repeated below.

*Hypothesis 1b*: when learning a new word expressing a complex meaning with meaning components M_*A*_ (initial state) and M_*B*_ (change of state), the meaning is construed as Ps(M_*A*_) and Assert(M_*B*_)

We also see that the “false” control animation behaves just like the animation only falsifying upward movement when it comes to its sensitivity to negation. This is surprising given that the “false” control animation falsifies both meaning components, including the one seemingly construed as presuppositional (movement from red). That is, in the presence of negation this animation should be a case of presupposition failure (the assertion is true as “not wugging” = downward movement). However, contrary to expectations, what we see is relatively low CB choices. Possible explanations for this include that negation is construed as “meta-linguistic” and that the presupposition is locally accommodated, both mechanisms allowing for the presupposition to be visible to negation (“the circle did not WUG as it never started from red”). The question is why the same option is not available for animations where upward movement is verified. The pattern could be an indication that the red line is truly interpreted as a precondition, i.e., moving downward could be result of not starting from red (just as not smoking can be the result of never starting). However, upward movement requires starting from red (just as smoking requires starting). Another possible explanation behind this pattern (also brought up by three anonymous reviewers) is that a conjunctive analysis of *wug* as “move upward (and) from red” was entertained. The fact that the two meaning components (conjuncts) are sensitive to negation to varying degrees might be rooted in the fact that one is construed as a modifier/adjunct, and that for this reason it is more likely to be backgrounded/“non-at-issue” content. While this is a possibility, the question becomes why such an analysis is spontaneously adopted by participants. A *post-hoc* analysis of individual patterns of responses generated no additional insights in that regard; see Supplementary Materials for details.^5^ A clear categorization of participants was not justified by the data; rather, response behavior varied within participants. Overall, it is clear that the data from embedding under negation alone are insufficient to identify presuppositional behavior. We thus looked at additional diagnostics and types of embedding in Experiments 2 and 3.

### Experiment 2

The aim of Experiment 2 was to test whether the triggering algorithm suggested by Experiment 1 can be overridden. To do so, we placed the participants in different groups and used negation-based training to indirectly teach them either that upward movement is part of the main assertive component of “wugging”, or that starting from the red line is.

#### Method

##### Procedure and materials

Experiment 2 followed the same basic procedure as Experiment 1. Participants were given the same instructions and were taught the meaning of *wug* by watching animations. Next, they were tested on their own usage of the word. Unlike Experiment 1, the teaching phase contained not only positive instances of “wugging”, but also negative ones. That is, participants saw negated sentence like (10) also during training.

(10) . The green circle does not wug.

We put participants in two different training groups in Experiment 2. The “canonical” group was trained with negation to construe upward movement as the assertion. That is, they were taught that “not wugging” is downward movement from the red line. This was done by showing them animations where the circle moved downwards from the red line with the sentence in (10). The “non-canonical” group was trained with negation to interpret starting from the red line as the assertion. Participants in this group were shown animations where the circle moved upwards but *not* from the red line with the sentence in (10). Participants in each group were shown 4 cases of “wugging” and 4 cases of “not wugging” during training. The critical, control and filler items used in the testing phase were the same as for Experiment 1.

##### Design

We manipulated three factors in Experiment 2; the between-subjects factor training group with two levels (non-canonical/canonical), and the same two factors within subjects as before: sentence polarity (positive/negative) and animation type (upward—from red, upward—not from red, not upward—from red, not upward—not from red).

##### Participants

Seventy-three participants (native speakers of English who did not participate in Experiment 1) participated in Experiment 2 through Prolific. They received 7,50GBP per hour compensation. Three of them were excluded from the analysis due to them responding incorrectly to more than 50% of true target animations (“upward—from red”). We analyzed data from 70 participants.

#### Hypotheses and predictions

The question addressed in Experiment 2 is: can any meaning component of a new complex lexical item be construed as assertion/presupposition by providing negation-based evidence? Put differently, if there is a triggering mechanism, how does training with relevant input interact with this mechanism (e.g., can it override it fully)? Investigating this question may help ascertain the strength of the triggering mechanism.

For contextual views, visibility to operators like negation is a property of “at-issue” content. They thus predict that negation based training should affect which parts of meaning come out as projective; see Hypothesis 2a below. Conceptual theories predict there to be a robust triggering algorithm, rooted in conceptual preferences. They thus predict that overwriting it should be hard; see Hypothesis 2b below.

*Hypothesis 2a*: any meaning component of a new complex meaning M with meaning components M_*A*_ (initial state) and M_*B*_ (change of state) can be construed as assertion after training with negation
*Hypothesis 2b*: for a new complex meaning M with meaning components M_*A*_ (initial state) and M_*B*_ (change of state), training with negation cannot override the default output of the triggering mechanism construing M_*A*_ as presuppositional

To test for an effect of training group, we look at the three-way interaction between animation type, sentence polarity and training group. If training leads to different interpretations regarding what the assertion/presupposition is, we expect this interaction to be significant. If H2a holds—consistent with contextual views—“upward” movement will be construed as assertion by the canonical group but as presupposition by the non-canonical group. Conversely, the non-canonical group should construe “from red” as asserted (since it is visible to negation according to training), whereas upward movement should show presuppositional behavior. The two critical animation groups should show distinct patterns, resulting in a three-way interaction.

If H2b holds—consistent with conceptual views—the initial state should be harder to construe as asserted meaning. Irrespective of training, participants should still construe the initial state as presuppositional. That is, there should be no three-way interaction.

One concern regarding the analysis of Experiment 2 is that CB choices for critical target animations might be affected by which of the two target animations participants saw in training. That is, the choice of CB might be driven by the animation simply not having been seen before, which should reduce certainty that this is an instance of “wugging”. To make sure that the asymmetry of what was novel for different groups (i.e., animations not seen in training) is not what is carrying the interaction, we look at the interaction between animation condition and novelty (coded as a dummy variable). If these two interact, with some animations being affected by novelty, the interaction with group will be harder to interpret. That is, the interaction could be carried by participants overall choosing more CBs for animations never seen before. This is asymmetric for the two groups, as target A (falsifying upward movement) is never seen by the “non-canonical” group whereas target B (falsifying movement from red) is never seen by the “canonical” group.

#### Analysis and results

**Fig. 5:**
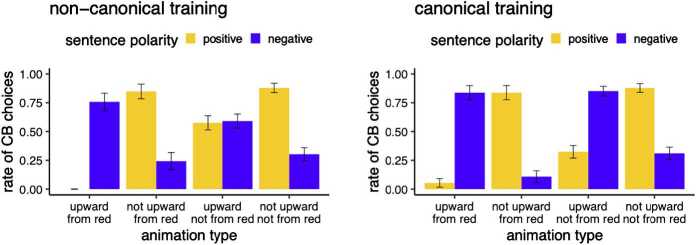
Exp. 2: mean rate of covered box choices by sentence polarity and animation condition for the two groups of participants under different training regime. Error bars indicate the standard error. **Left:** non-canonical training involved showing “not wugging” as upward movement not starting from the red line. **Right:** canonical training involved showing “not wugging” as downward movement starting from the red line.

We followed the same procedure for analysis as described for Experiment 1.^6^ The results of Experiment 2 are summarized in Fig. 5. Overall, the pattern for the group trained to construe “from red” as at issue/asserted looks very similar to the pattern we observed in Experiment 1, where participants were not trained with negation at all. For the animation conditions the non-canonical group was trained with, we see an equal amount of covered box choices with positive and negative sentence polarity. Participants receiving “canonical” training, however, showed a different behavior. The animation conditions participants were trained with show high covered box choices with negation and low covered box choices without negation, just like false controls. To test the different variants of Hypothesis 2, we first looked at the three-way interaction between training group, animation type, and sentence polarity. The model comparison between a model including the three-way interaction term (button_pressed ∼ cond ∗ negation ∗ group + (1 | subjectId)) and a model without the three way interaction term (button_pressed ∼ cond ∗ negation ∗ group - cond:negation:group + (1 | subjectId)) via log likelihood ratio tests revealed that the interaction term is justified ($\chi^{2}(3)=16.7$, *p*<.001). We also coded a dummy variable novelty which indicates whether an animation was new to participants in the testing phase. This variable does not show an interaction with animation condition and negation. This suggests that the effect we see is not due to the fact that the animations are novel.

To investigate what the interaction is carried by, we looked at the interaction of sentence polarity with animation type per training group. For the canonical group (negative instances of “wugging” are downward movement), the model comparison via log likelihood ratio tests revealed this interaction to be justified ($\chi^{2}(3)=41.582$, *p*<.0001). We then looked at least square means contrasts with the emmeans package in R (Bonferroni-corrected for multiple comparisons). We saw that there is a difference between positive and negative sentences for all animation conditions; see Table 4. That is, we see a reversal of the CB choice pattern with negation for downward movement, regardless of what the initial state was (red line/not red line). For the non-canonical group, trained with stimuli that went against the triggering algorithm (negative instances of “wugging” are not starting from the red line), we also find the interaction term to be justified ($\chi^{2}(3)=40.434$, *p*<.0001). Looking at contrasts, we see that there is a difference between negative and positive sentence polarity for the target animation condition “not upward—from red” (*β̂* = −2.8622, SE = 0.633, p < .0001). However, crucially, there was no difference between the CB choices for negative and positive sentences when the animation condition was “upward—not from red” (*β̂* = 0.0623, SE = 0.353, *p* = 1). That is, the statistical pattern we see for the “non-canonical” group is the same as the one in Experiment 1 where participants did not receive negation-based training.

**Table 4:** Exp. 2: Pairwise comparison output of contrasts for the canonical training group, calculated based on least square means with the emmeans package in *R*

cond	Contrast	*β̂*	SE	z.ratio	p.value
upward—from red	neg - pos	4.504	0.853	5.281	*p* <.0001
not upward—from red	neg - pos	−3.752	0.692	−5.421	*p* <.0001
upward—not from red	neg - pos	2.479	0.410	6.041	*p* <.00011
not upward—not from red	neg - pos	−2.773	0.435	−6.370	*p* <.0001

#### Discussion

The results of Experiment suggest that what meanings are considered presupposed versus asserted by participants depended on the training they received with negation being in line with the conceptual bias or being inconsistent with the conceptual bias. More specifically, we see that negation-based training against the bias towards initial states being presupposed meaning does not change the basic pattern we found in Experiment 1. Even when training with negation suggested that the initial state (being on the red line) was “at-issue” content, participants are more likely to construe the initial state as “non-at-issue”/presupposed, as shown by the relevant animation condition (‘upward—not from red’) leading to equal rates of CB choices with negative and positive sentences. We take this to be evidence for H2b repeated below.

*Hypothesis 2b*: For a new complex meaning M with meaning components M_*A*_ (initial state) and M_*B*_ (change of state), training with negation cannot override the default output of the triggering mechanism construing M_*A*_ as presuppositional.

Just like in Experiment 1, we thus find that the two meaning components of the new complex meaning *wug* behave differently under negation for the “non-canonical” group, with one part of the meaning (the initial state) showing a pattern that is more in line with an analysis as a presupposition, the other displaying behavior more in line with an analysis as assertion. However, we also see that training in line *with* the triggering algorithm does not show the pattern we observed in Experiment 1. Instead, the pattern for the canonical training group is now consistent with participants not considering “from the red line” as part of the meaning of *wug* anymore. That is, the animation where (only) movement from red is falsified behaves like “true” control animations. If a conjunctive analysis had been entertained by participants, both critical animations should behave like the “false” control animation. If the initial state was still perceived as presuppositional, the same patterns as for Experiment 1 and the “non-canonical” group would have been expected. Perceptually speaking, the red line is very salient and kept constant in training as the starting point for the canonical group. It is thus interesting that negation alone can guide attention away from this possible meaning component of “wugging”.

This finding is not predicted by any theory of presupposition triggering on the market. There is, however, evidence that presuppositions CAN be ignored for the sake of verifying the assertion for hard triggers like *again* (Tiemann et al. 2015). We speculate that this possibility might be available due to the special pragmatics involved when training with negation. If this strategy were generally available, it would be problematic for the paradigm we suggest here, but see also our Discussion of Experiment 3 below. What is relevant for the current discussion is that negation based training in the *opposite* direction does not have a similar effect, i.e., it does not lead participants to ignore upward movement, nor does upward movement display behavior which is in line with it being presuppositional. This asymmetry is indicative of conceptual biases in the triggering of presuppositions associated with new words. We, again, looked at individual participants’ behavior to gain additional insights; see Supplementary Materials for details. We see that more participants fall into the category of presupposing movement from the red line or ignoring the red line with canonical training. We also see that, with non-canonical training, participants are more likely to adopt a conjunctive analysis and are less likely to simply ignore the red line. These explorations tentatively suggest that ignoring movement from red versus considering it to be part of the assertion is not driving the overall effect we observe alone. More data are needed to look into the issue of what might influence individual participants’ behavior, especially regarding when they consider certain components to be part of a meaning at all.

The results of Experiment 1 and 2 also tentatively suggest that a translation into conjunction (“moves upward (and) from red”) is not the default strategy. If this were the case, we would predict that the critical animation falsifying “from red” behaves like a false control. To make sense of the fact that the critical animation condition falsifying “from red” does not behave like false controls under the conjunctive analysis, one needs to assume that something, e.g., focus, makes the “upward” component less salient. In the presence of focus, the prepositional phrase could potentially be targeted by negation. That is, participants would need to structurally analyze the negated sentence as “The circle does not move upward FROM RED”. While this is certainly an option, we do not consider it to be a plausible one for two reasons. One, this meta-linguistic use of negation is not very frequent. Two, without contextual pressure putting focus on the prepositional phrase is a marked option. One goal of Experiment 3, described in the next section, was to avoid additional complications added by negation, especially its sensitivity to focus placement. We provide evidence from quantificational elements speaking for an interpretation of the overall results as showing that initial states are more likely to be considered presupposed or “non-at-issue”.

### Experiment 3

The goal of Experiment 3 was to extend the inquiry to more complex and diagnostic cases of presuppositions: projection out of the scope of quantified expressions. There is a debate in the theoretical and experimental literature alike whether presuppositions project universally from the scope of quantifiers (Heim 1983; Schlenker 2008, 2009), see the universal presuppositions of (11) and (12) in (11-a) and (12-a), respectively, or project existentially, see the presuppositions in (11-b) and (12-b) (Beaver 1992, 1994, 2001).

(11)

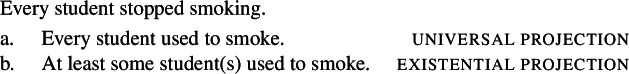

(12)
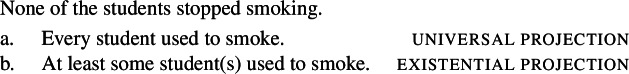

Whether a given quantifier allows for existential projection has been tied to its semantic properties (Heim 1983; Schlenker 2008), as well as contextual information (Beaver 2001; Charlow 2009). Support for the view that the quantifier itself plays a role for the level of projection comes from experimental explorations of the issue, which suggest that both existential and universal projection exist to varying degrees, depending on the quantifier (Chemla 2009; Tiemann 2014; Geurts and van Tiel 2016). As discussed in Sect. 3 above, different levels of projection have been observed previously for *none* and *every* using the covered box paradigm (Creemers et al. 2018; Zehr et al. 2016). If the new word meaning people acquire has a presuppositional component, we expect to see at least existential projection of this meaning component. If we find evidence for universal projection and a difference between the two projection levels, we have an additional, more specific pointer to presuppositional behavior than the one suggested by behavior under negation.

#### Method

##### Procedure

Overall, Experiment 3 followed the same logic as used in Experiments 1 and 2. In the first phase, participants were taught a new word *wug* through simple animations. They first read the same simple instructions as participants in the first two experiments. There was only one training group in Experiment 3. Training was based on negation, that is, participants saw sentences with and without negation in the training phase. Furthermore, training was always canonical, that is, participants were taught that “wugging” is upward movement from the red line, whereas “not wugging” is downward movement from the red line (each occurred 4 times).

**Fig. 6:**
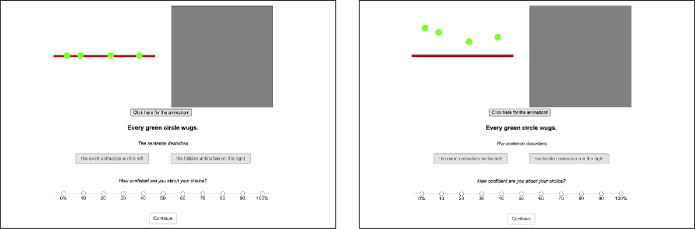
**Left:** Initial state displayed after the “Click here for the animation”-button was pressed. **Right:** Result state of the animation displayed. The animation depicted is a case of a true control condition with the quantifier *every*.

As before, participants saw an overt and hidden animation in the testing phase (see a sample trial in Fig. 6). They were asked to choose the overt animation if it is what the sentence describes. Otherwise, they were asked to choose the covered animation. In addition, their task was to rate how confident they were while making their decision on a scale from 0% confident to 100% confident. The idea was that this would provide an additional measure of presuppositionality. Specifically, we speculated that if people chose the overt animation with violated existential or universal presuppositions, they would at least be hesitant about it, which in turn should affect their confidence level. We also included number of clicks on the “Show me the animation”-button as an additional measure of uncertainty.

##### Materials

The testing phase contained critical sentences with quantifiers *every* or *none*; see (13).

(13)
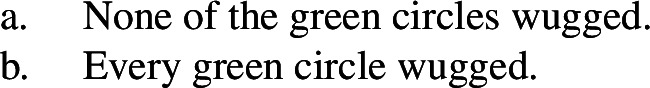

They were paired with different animation types that falsified/verified the asserted part, and falsified/verified the universal or existentially projecting presupposition; see Table 5.

**Table 5:**
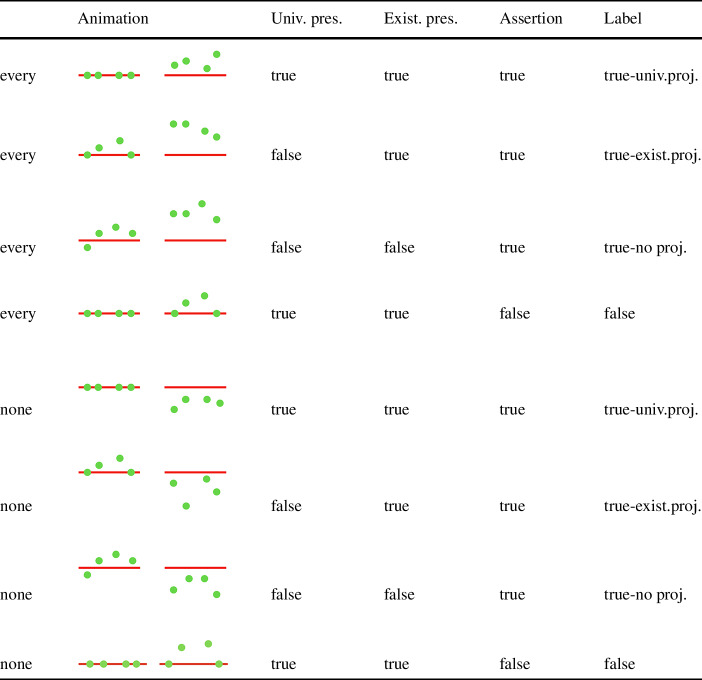
Initial and result state of different critical animation conditions and the readings they correspond to for each quantifier.

Each of these critical conditions appeared four times in the experiment (=24 critical targets in total). In addition, we included three false and three true control cases with the quantifier “some”. For the true cases with “some”, we used the same animations as for the false *every* cases with universally projecting presuppositions (line 4 in Table 5). For the false cases with “some”, we used the same animations as for *none* cases where the assertion is false and universal presupposition is true (line 8 in Table 5). The idea was to add more variability and avoid biases for certain animation conditions. We also included six control cases with simple sentences (“The green circle {does not} wug(s)”) in combination with animations they were trained with (upwards or downwards movement from the red line). The resulting 36 trials were pseudo-randomized. The participants were first shown simple sentences and then four true quantified sentences (two per quantifier). After that, the rest of trial followed in fully randomized order.

##### Design

We manipulated two factors, the within-subjects factor quantifier with two levels (every/none), and the within-subjects factor projection level given by the animation (three levels: true-universal/true-existential/true-no projection). The dependent variables were the rate of covered box choices, level of confidence, and the number of repeated clicks on the “Click here for the animation”-button.

##### Participants

We tested 100 participants through Prolific. They were all native speakers of English (first language) and did not participate in any previous experiments on presupposition triggering that we conducted. They received 7,50GBP per hour compensation. We excluded three participants who responded incorrectly to more than 50% of the simple control cases they were trained on. We thus looked at data from 97 participants.

#### Predictions

According to all views, the negation based training participants received in Experiment 3 should clearly point to upward movement as asserted, at-issue content. However, theories make different predictions for the “from red” component of “wugging”. If starting from the red line is construed as presuppositional given canonical training—in line with conceptual views—we predict all dependent measures to be sensitive to different levels of projection. That is, animations violating existential or universal readings should give rise to some CB choices, in line with previous findings, but not behave like “false” controls. Moreover, based on previous results, we also expect to find difference between quantifiers.

It is less clear what the predictions of contextual views are for the projection of “non-at-issue” content from quantificational statements. As far as we can tell, the placement of focus should have bearing on the presence of different levels of projection, as the meaning component “from red” remains an entailment and should thus still visible to quantificational operators. As a result, any animations falsifying the universal reading or the existential reading should behave like “false” controls. Even when making the (controversial) assumption that background information can show presupposition-like behavior (Geurts and van der Sandt 1997), it is unclear why different quantifiers would be sensitive to given vs. focused information to varying degrees. It is also not clear how different levels of projection (existential vs. universal) would be accounted for.

A third possibility, given the findings of Experiment 2, is that “from red” is simply not considered to be part of the meaning of *wug* at all. Crucially, there should be also no difference between projection levels if that is the case. The animations “true-no-projection” and “true-existential-projection” should behave like “true” controls in that case.

#### Analysis and results

**Fig. 7:**
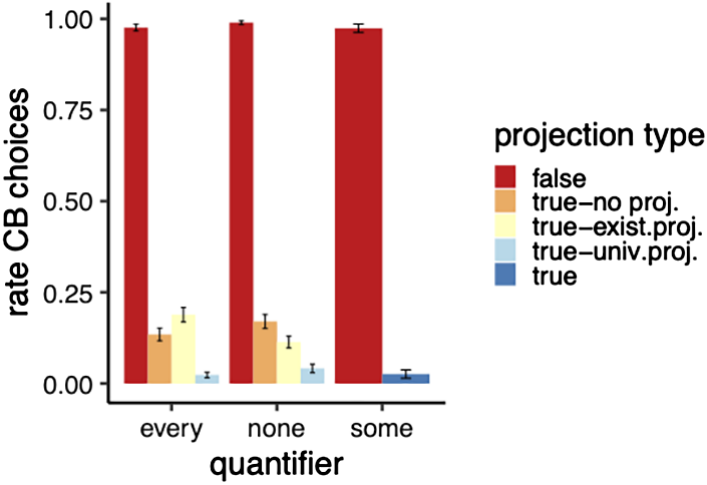
Exp. 3: Mean rate of CB choices by projection level and quantifier. Error bars indicates the standard error. The *some* control sentences were only presented with animations where all circles moved from the red line (universal projection) upwards (true control) or downwards (false control). For the critical sentence types with *none* or *every*, true sentences satisfied different presuppositions; see Table 5

The first measure we looked at was the rate of CB choices by quantifier and projection level, see Fig. 7. We see CB choices for conditions violating the existential or universal presuppositions. We also observe small differences between quantifiers.

Using generalized linear mixed effect models, we tested for an interaction between quantifier and projection level with a model comparison through log-likelihood ratio tests, comparing a model with the interaction term (button_response ∼ projection ∗ quantifier + (1 | subjectId) + (1 | id)) to a model without (button_response ∼ projection + quantifier + (1 | subjectId) + (1 | id)). It revealed that the interaction term is justified ($\chi^{2}(2)=9.2294$, *p*<.01), suggesting that the two quantifiers behave differently with regard to how much they are sensitive to different levels of projection. We did Bonferroni-corrected pairwise comparisons between projection levels per each quantifier; see Table 6. They reveal that, for both quantifiers, animations verifying existential projection differ from animations verifying universal projection (“true-existential” vs. “true-universal”). We also have evidence that the case where neither existential nor universal presuppositions were satisfied (“true-no-projection”) differed from cases where universal presupposition was satisfied (“true-universal”). That is, overall we find evidence of universal projection for both quantifiers. However, for both quantifiers we see no contrast between “true-no projection” animations and “true-existential” animations, i.e., no evidence for existential projection.

**Table 6:** Exp. 3: Pairwise comparison output of contrasts calculated based on least square means calculated with the emmeans package in *R*

Qua	Contrast	*β̂*	SE	z.ratio	p.value
every	existential - no projection	−0.600	0.309	−1.941	*p* =.4698
every	existential - universal	−3.038	0.692	−6.528	*p* <.0001
every	no projection - universal	2.438	0.410	−5.200	*p* <.0001
no	existential - no projection	0.682	0.318	−1.941	*p* =.2874
no	existential - universal	−1.429	0.692	0.438	*p* <.02
no	no projection - universal	−2.112	0.410	0.431	*p* <.0001

**Fig. 8:**
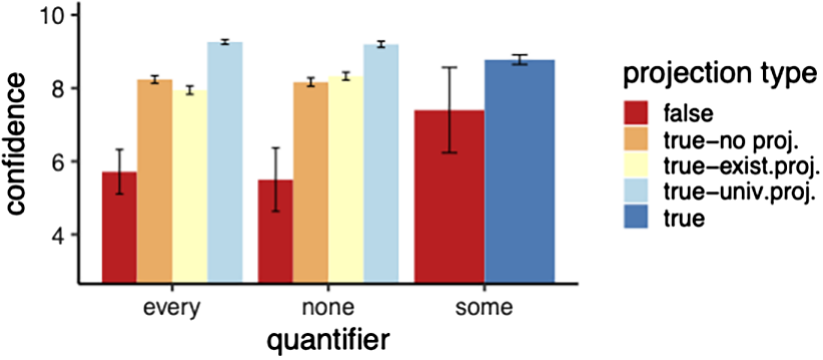
Exp. 3: Mean confidence ratings by projection level and quantifier (for overt picture choices). Error bars indicate the standard error. The “some” control sentences were only presented with animations where all circles moved from the red line (universal projection). For the critical sentence types with *none* or *every* true sentences satisfied different presuppositions, see Table 5

Second, we moved to confidence ratings when the choice was the overt picture (=majority response), see the results in Fig. 8. We see lower confidence for existential and no projection cases, and see no differences between quantifiers.

For the confidence rating data, we ran a cumulative link model analysis using the clmm function of the ordinal package in *R* to test for an interaction projection level with quantifier. Again, we compared a model with the interaction term (responses ∼ projection ∗ quantifier + (1 + projection | subjectId)) to a model without it (responses ∼ projection + quantifier + (1 + projection | subjectId)). A log likelihood ratio test revealed that the interaction term is justified ($\chi^{2}(2)=16.541$, *p*<.001). We then again did a pairwise comparison between projection levels for each quantifier (Bonferroni corrected); see Table 7.

**Table 7:** Exp. 3: Pairwise comparison output of contrasts calculated based on least square means calculated with the emmeans package in *R*

Qua	Contrast	*β̂*	SE	z.ratio	p.value
every	existential - no projection	−0.644	0.181	−3.566	*p* <.01
every	existential - universal	−4.185	0.501	−8.358	*p* <.0001
every	no projection - universal	−3.54	0.480	−7.379	*p* <.0001
no	existential - no projection	0.254	0.177	1.438	*p* = 1
no	existential - universal	−3.454	00.502	−6.887	*p* <.02
no	no projection - universal	−3.709	0.485	−7.642	*p* <.0001

The universal conditions, satisfying both the existential and universal projection readings, differ from conditions only satisfying the existential reading and from conditions where both the existential and universal presupposition is false (“true-no-projection”). This holds the same for both quantifiers. We only have evidence for existential projection for the quantifier *every*, as the “true-no-projection” animations differ from animations violating the universal presupposition (“true-existential”). This effect is numerically very small.

**Fig. 9:**
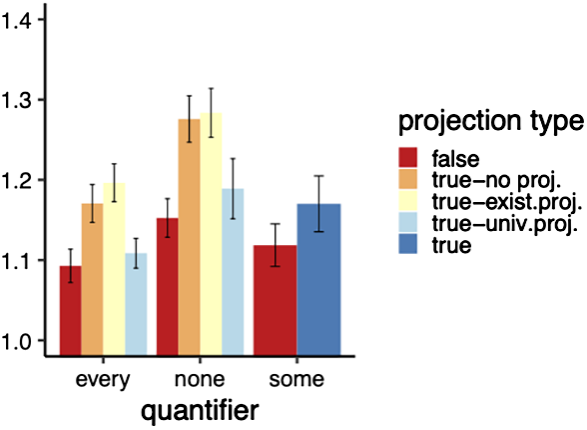
Exp. 3: Mean number of clicks on animation by projection level and quantifier. Error bars indicate the standard error. The *some* control sentences were only presented with animations where all circles moved from red (universal projection) upwards (true control) or downwards (false control). For the critical sentence types with *none* or *every* true sentences satisfied different presuppositions, see Table 5

Finally, we looked at the number of repeated clicks on the “Click here for the animation”-button; see the results in Fig. 9. First, to see whether a Poisson regression or a negative binomial model is more appropriate, we tested for overdispersion using the od.Test function of the pscl package in R. It performs a log likelihood ratio test comparing the Poisson model against a negative binomial model. The difference in likelihood between the two modals was significant (*p* <.0001), suggesting that a negative binomial model is more appropriate. We thus fit a negative binomial regression model using the glm.nb function of the MASS package in R. To test for an interaction between quantifier and projection level, we compared a model with the interaction term (glm.nb(repeats ∼ projection ∗ quantifier, data = critical)) to a model without it (glm.nb(repeats ∼ projection + quantifier, data = critical)). This interaction did not turn out to be significant; we thus did no further comparisons.

#### Discussion

Further evidence for the existence of conceptual biases in presupposition triggering comes from the usage of new word meanings in quantificational statements. We see that the initial state of the newly acquired change-of-state verb *wug* is not treated as part of the assertion, but rather displays the subtle behavior of presuppositions in these environments. Animation conditions violating the universal or existential presupposition give rise to different rates of CB choices from “false” controls. Moreover, accepting the animation showing failure of the existential or universal presupposition leads to lower confidence in judgments. These two findings are *prima facie* harder to reconcile with contextual views, where the initial state is a “non-at-issue” entailment and as such visible to the quantifier. More research on the interaction of quantifiers with “non-at-issue” content and focus is needed to fully assess the predictions of these accounts, however. Importantly, as opposed to the results of Experiment 2, we find no evidence for participants simply ignoring the red line. That is, we do see that, when the “from red” component does not universally project, people choose the CB around 15% of the time, which is different from the at floor rates of CB choices with true controls. We speculate that explicitly training participants with negation and then using negation as a test case for projection may have masked presuppositional behavior. The CB rates we observe overall seem quite low. It is, however, important to keep in mind that CB choices for violated existential and universal presupposition were as low as 25% with existing words (Creemers et al. 2018). The fact that we do not find clear evidence for existential projection for universal quantifiers is in line with previous findings (Zehr et al. 2016) (but see Zehr et al. 2015 for different results). An extension to existential quantifiers might give additional insights into the question whether the paradigm is also suited to investigating more intricate theoretical questions regarding projection through quantifiers. Given that the only effect of existential projection we see is small and only visible in one measure, we are hesitant to draw any theoretical conclusions for now. Overall, the findings are also a further validation of the methodology suggested. We see evidence from three different measures that the meaning component that refers to the initial state (starting from the red line) displays presuppositional behavior. Our data suggest that a combination of different embedding environments and dependent measures is needed to clearly identify presuppositional behavior.

## General discussion

Our results provide new important insights into the mechanisms behind presupposition triggering. They also point to a new fruitful methodology for investigating the factors influencing what entailments are more likely to become presuppositional or “non-at-issue” (projective) content.

First, with limited training input based on animations, participants infer that the initial state (“from red”) of a newly acquired change-of-state verb is presupposed or not “at-issue”. This is shown by the behavior of the newly learned word (i) under negation and (ii) in the scope of quantifiers. Regarding (i), a statement and its negation give rise to the same amount of CB choices in the relevant animation condition (“upward—not from red”). Regarding (ii), we have evidence for universal projection of this meaning component under the quantifiers *none* and *every* with two different measures. These patterns together are neither in line with a conjunctive analysis where both meaning components are part of the assertion, nor with any of these meaning components not being part of the meaning of *wug* at all, i.e., one meaning component being ignored.

Second, we have shown that there might be a conceptual bias for construing initial states as presuppositional which cannot be easily overridden. When trained with negation targeting the initial state (“from red”), some participants still construe the initial state as presuppositional.

On a methodological level, our findings thus suggest that a combination of dependent measures and types of embedding structures are needed to detect presuppositional behavior. Specifically, evidence from negation alone is inconclusive, possibly due to its interaction with focus and, relatedly, the possibility of it being interpreted meta-linguistically. Training with negation in Experiment 2 seemingly led to (some) participants completely ignoring the red line. However, in Experiment 3, where participants were also trained with negation, we still see projection with quantified expressions. It is thus clear that negation not only affects interpretation of *wug* but also that training with negation has different effects across dependent measures.

Our results align with recent findings on acquisition of presuppositions suggesting that at an early age kids identify presuppositions as imposing conditions on the context and act accordingly (Aravind et al. 2023). Even though input for children is also limited and, in addition, conflicting, learning that presuppositions must be satisfied in the context precedes them learning that presuppositions can be informative in some context by accommodation.

Our main findings are hard to reconcile with accounts of presupposition triggering that stress the role of lexical alternatives (Abusch 2002). Since no alternatives were given in our stimuli, deriving the presupposition from a disjunction of lexical alternatives seems difficult. To make the strong version of an alternative-based account work, one would have to assume that, e.g., “downward movement from red” is also lexicalized in the language of this experiment, and furthermore that “upward movement not from red” is not lexicalized. More likely, one would have to revert to a conceptual view of alternatives, in which alternatives are salient concepts, whether lexicalized or not (Chemla 2007; Buccola et al. 2021). Even then one would have to justify why “downward movement from red” is a more fundamental concept than “upward movement not starting from red”. If anything, this moves closer to a conceptual account of the triggering problem again.

A similar point can be made with regard to the perspective offered by theories working with contextual (focus) alternatives and questions (Simons et al. 2011; Tonhauser et al. 2018). The fact that the initial state was construed as presuppositional with only positive input is puzzling under this view. As mentioned above, the question becomes why one meaning component (change-of-state) should more readily be accepted as “at-issue” than “non-at-issue” when, at first glance, nothing in the input was indicative thereof. The most likely CQ raised by training is simply “Does the circle wug?” However, again, nothing in the input reveals whether this is a question about moving upward or moving from the red line. Two anonymous reviewers pointed out that a conjunctive analysis might have been entertained where one of the conjuncts carries focus (which would make that conjunct at-issue). Specifically, the idea is that *wug* is translated into “moves UPWARD (and) from the red line”. While this is a possibility, there are arguments against it in view of the data. One argument against this option is that this type of focus placement would not account for the projection pattern observed in the presence of quantifiers. Furthermore, even when this analysis is considered by participants, the issue once more becomes why one of the conjuncts is more likely to carry focal stress and thus mark the CQ. Factors might include the category (VP/PP), syntactic status and visual salience. Psychological research on the latter (mostly employing search paradigms) suggest that color is MORE salient than movement, however. More independent criteria for determining the CQ is needed to test the predictions of contextual theories with the suggested paradigm and extensions thereof. Another issue for contextual views is the fact that training with negation was not used to make predictions for what is at-issue, however. The input used in Experiment 2 made it clear what parts of the meaning are visible to operators. Nonetheless, participants treat the initial state as projective content in the non-canonical group, and not part of the meaning of *wug* in the canonical group. Furthermore, it is unclear to us at this point in how far “non-at-issue” content can be invisible to quantified expressions, as suggested by the fact that sentences with quantifiers are accepted at a high rate in visual scenarios allowing for only existential or no projection.

For now, both findings are in line with conceptual theories of presupposition triggering. For Abrusán (2011), the “moving upwards” part of “wugging” is about the topic time, whereas “starting from red” precedes the topic time. Since only entailments that are *not* about the topic time are presupposed, her triggering algorithm correctly predicts the pattern we see. Under Schlenker’s (2021b) view “starting from the red line”—the initial state—is more likely to be presupposed as it is temporally antecedent to “moving upward”. As a result, one is more likely to have antecedent beliefs about it.^7^ However, to fully test and confirm these theories more data are needed. Specifically, the paradigm should be extended to other types of change of state verbs and presupposition triggers. The hypothesis that temporal precedence matters, for example, could easily be tested by comparing initial versus result states with the current paradigm.

While our results at this point are easier to reconcile with some views on triggering than others, it is important to note that the findings are in line with a perspective where different factors that have been argued to be involved in presupposition triggering, including information structure and alternatives, interact. For example, conceptual biases and common lexical structures might be enmeshed with what likely CQs or conceptual alternative are in a given context. The concept of temporal *aboutness* discussed by Abrusán (2011), for example, is clearly related to what is currently “at issue”. Additional biases might also be constructed through the nature of the animations and visual set up. Rather than viewing the ideas just presented as competing a more promising route is to use the paradigm presented to investigate different factors involved in how different layers of meaning come about.

All three experiments speak to the success of the new methodology and establish further measures for presuppositional status. First, we demonstrate that the CB paradigm works in combination with word-learning tasks. Second, we showed that training against the predictions of a triggering algorithm can be used as an indicator for which meaning components are presupposed. Third, the findings of Experiment 3 suggest that the level of confidence when making a choice are indicators of presuppositional status. It is furthermore a revealing measure regarding how reluctant participants are to choose a covered box versus an overt picture violating different parts of meaning, thereby granting more insights into the paradigm. We suggest that the methodology developed can and should be used to further test existing theories, especially given some limitations of the data presented. This regards individual participants’ behavior but also the type of presupposition trigger tested. Extension to other (non-verbal) types of triggers might be difficult due to the visual representation being much harder, or due to the fact that inventing new words for new closed class vocabulary such as determiners might be more restricted.

Stepping back, our results dovetail with recent attempts to display a triggering algorithm in action by way of novel pro-speech gestures and visual animations. These attempts treated these gestures and visual animations as new “words”, whose semantic content is divided on the fly between an at-issue and a presuppositional component. One might worry that these expressions look nothing like words, and might even trigger presuppositions because they are mentally translated into normal (but presuppositional) words. Our experiment also involves animations, but with a completely different role, since they display situations that affect the truth conditions of our target expressions. Since these are normal words, it is implausible that they are understood by way of translation into other words. It is thus reassuring that they yield the very same result as the iconic expressions that were studied earlier, and strengthen the case for the existence of a triggering algorithm. We concede that multiple factors might be involved in predicting what meaning components end up presupposed versus asserted, and that it might be dependent on the exact nature of the complex meaning that is acquired. Our data offer a starting point for investigating the issue further, and extending our paradigm to other types of verbal presupposition triggers and animation types.

## Data Availability

The original data and analysis script can be found under the following osf link: https://osf.io/es74b/?view_only=2725a5622e4a442287cd2aba63ef0827.

## Footnotes

1 The bivalent truth-conditional content of a propositional expression is the bi-partition between situations the expression holds vs. does not hold of. The notion can be extended for any expression whose type “ends in t”: its bivalent content is the bi-partition between inputs it does vs. does not hold of.

2 For alternative proposals involving the use of alternatives where the relevant parts of meanings are entailments not presuppositions, see Chemla (n.d.) and Romoli (2011).

3 Less relevant for the current discussion is that “at-issueness” as well as “projection” are considered gradient notions. The degree to which a component is “at-issue” should match its ability to project (Tonhauser et al. 2018).

4 For more details on the analysis, see Supplementary Materials.

5 Many thanks to an anonymous reviewer for thorough guidance on the issue.

6 For more details on the analysis, see Supplementary Materials.

7 To fully test the predictions of Schlenker’s theory for a movement of object x from A to B, one would need to assess the intuitive probability that upon learning that x moved from A to B, one had preexisting knowledge that x was initially in A (in addition, one would need to establish the probability threshold at which entailments that satisfy this condition become presupposed). Since this would be a separate project, we follow Schlenker (2021b) in taking his theory to make the same general predictions as Abrusàn’s relative to change-of-stage constructions.
